# Direct Perforator Anastomosis of Free ALT Flap in Massive Weight Loss Due to Increased Size of Vessels

**Published:** 2017-06-14

**Authors:** Amra Kuc, Kathryn King, Justin Daggett, Deniz Dayicioglu

**Affiliations:** ^a^University of South Florida Morsani College of Medicine, Tampa; ^b^Division of Plastic Surgery, Department of Surgery, University of South Florida Morsani College of Medicine, Tampa

**Keywords:** anterolateral thigh flap, massive weight loss, perforators, distal 1/3 defect reconstruction, Gustilo Grade 3B tibial fracture, free flap

## DESCRIPTION

A 67-year-old male patient with a history of massive weight loss (MWL) presented with a left lower extremity wound (30 × 15 cm) with an exposed tibiofibular joint following a motorcycle accident. He underwent reconstruction with a left anterolateral thigh (ALT) fasciocutaneous free flap. The size of perforators of the ALT flap allowed for their use as direct donor vessels.

## QUESTIONS

1. **What are reconstructive options for distal third leg defects?**
2. **What is the anatomy of the anterolateral thigh (ALT) flap and perforator vessels?**
3. **What are predictors of vessel size relating to body mass index (BMI)?**
4. **What were some considerations in free flap design in our MWL patient?**

## DISCUSSION

Distal third leg defect reconstruction represents a frequent challenge for the reconstructive surgeon. The paucity of tissue limits local options to adipofascial turnover flaps and perforator-based propeller flaps. While perforator-pedicled propeller flaps can be good options for reconstruction of smaller defects, free flaps are typically required for larger defects, degloving injuries, and when composite reconstruction is needed.^1^ Common donor sites for free flaps are gracilis, latissimus dorsi, and ALT/vastus lateralis flaps, depending on the size of the defect and soft tissue components needed.^2^ Nevertheless, the goal is to perform the least invasive surgery possible. Typically, limitations of vessel size lead the surgeon to dissect proximally to the source vessels in order to obtain adequate vessel caliber. However, it has been observed that in patients with higher BMI and in MWL patients, the increased vessel diameter may allow surgeons to perform a less invasive surgery by using vessels that are normally too small for anastomosis.

The ALT free flap is a workhorse flap for reconstruction of the upper and lower extremities, skull base, trunk, head, and neck.^3^ The vascular pedicle is on average 16 cm in length, with the artery diameter of 2.1 mm and vein diameter of 2.6 mm. The vascular supply to the pedicle in 90% of patients is from perforators of the descending branch of the lateral circumflex femoral artery (Type I). In Type II (4%), a single perforator descends from the transverse branch of the lateral circumflex femoris artery, extending though the vastus lateralis. A Type III perforator (4%) is a direct branch of the profunda femoris, descending though the rectus femoris. Patients have on average 2.04 perforators, ranging from 0-3.^4^^,^^5^

When planning free flap reconstruction, vessel size should be considered. Vessel size is increased in patients with increased BMI, but does the size remain after weight loss? One study assessed superficial inferior epigastric vessel (SIEA) size and anatomical features in the MWL population. The results indicated vessel size is positively correlated to pannus weight, current BMI, and maximum BMI, with the maximum BMI being the strongest predictor of SIEA presence and usability. The SIEA was usable in 52% of MWL patients.^6^ Lower extremity vessel size in MWL patients has not been documented extensively.

We present a 67-year-old man with a history of 205-lb weight loss following gastric bypass surgery. His maximum BMI was 49.9; BMI at the time of surgery was 25.0. He presented to us with an open left distal one-third tibial and fibular Gustilo grade 3B fracture with a corresponding wound following a motorcycle accident. Soft tissue reconstruction commenced following adequate debridement and boney fixation. Given the size of the defect and location within the distal 1/3rd of the leg, the decision was made to proceed with a free ALT flap for reconstruction. After separating the vastus lateralis muscle fibers we encountered two major perforators of significant vessel caliber. The decision was made to use these for arterial anastomosis rather than dissect more proximally to harvest the lateral circumflex femoral artery. We then performed the arterial anastomosis using 8/0 nylon sutures and 3-mm coupler for venous coupling. A small size discrepancy of the artery was present. After good flow through the flap was observed and flap inset started, the patient had external fixators placed by our orthopedic colleagues. Patient had an uncomplicated recovery.

In conclusion, while a history of a high BMI, even in MWL, may place a patient at increased rates of postoperative complications, increased vessel size may allow for unique considerations such as the use of perforators for anastomosis.^7^

## Figures and Tables

**Figure 1:**
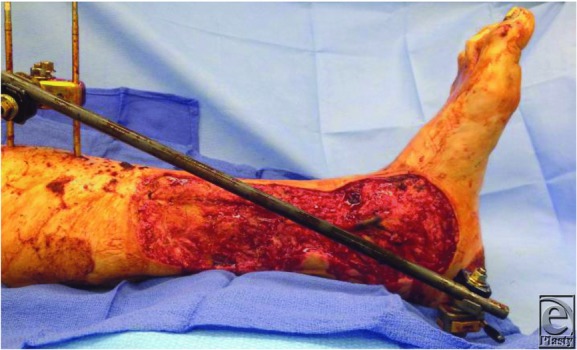
Left distal 1/3 defect with exposed tibial fracture site.

**Figure 2:**
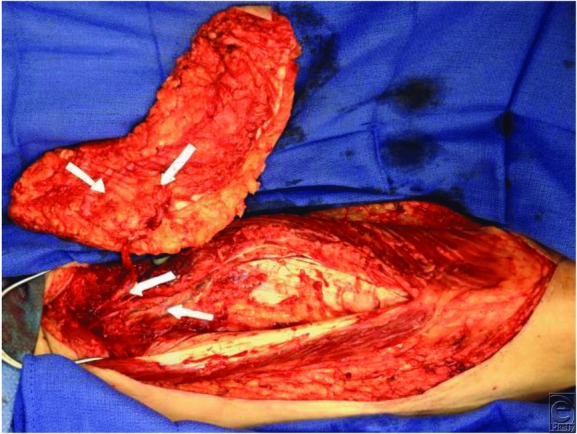
Intraoperative view of perforator dissection for the left anterolateral thigh flap. Arrows indicate (from bottom to top) the descending branch of the lateral circumflex femoral artery, intramuscular perforator (dissected), and superficial suprafascial branches of intramuscular perforator.

**Figure 3:**
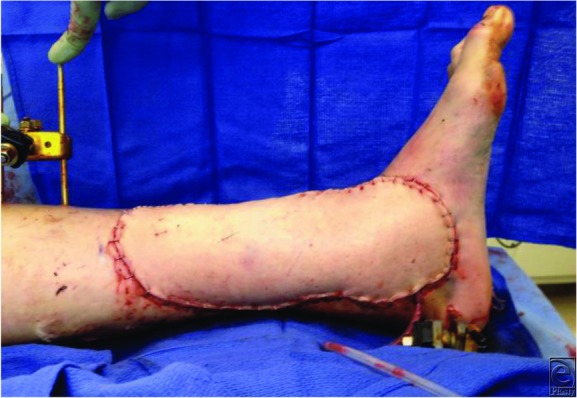
Final flap inset of the fasciocutaneous anterolateral thigh flap.

**Figure 4:**
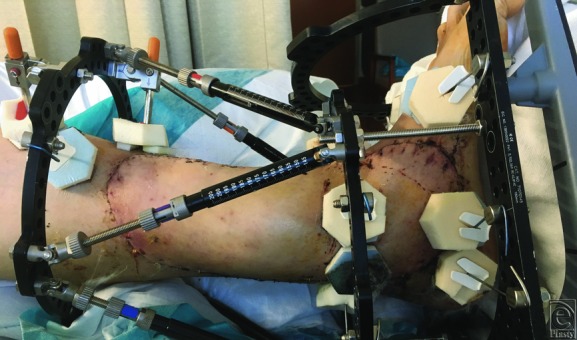
Two weeks postoperatively.
